# Rapid reuptake of granzyme B leads to emperitosis: an apoptotic cell-in-cell death of immune killer cells inside tumor cells

**DOI:** 10.1038/cddis.2013.352

**Published:** 2013-10-10

**Authors:** S Wang, M-f He, Y-h Chen, M-y Wang, X-m Yu, J Bai, H-y Zhu, Y-y Wang, H Zhao, Q Mei, J Nie, J Ma, J-f Wang, Q Wen, L Ma, Y Wang, X-n Wang

**Affiliations:** 1 The Institute of Life Sciences, Chinese PLA General Hospital and South China University of Technology, The State Key Laboratory of Kidney Disease, Beijing 100853, China, The Provincial Key Laboratory of Biotechnology, Guangzhou 510006, China; 2 The Second Affiliated Hospital, Guangzhou Medical University, Guangzhou 510260, China; 3 School of Ophthalmology and Optometry, Wenzhou Medical University, Wenzhou 325000, China; 4 Shanghai Institute of Immunology, Department of Immunology and Microbiology, Institute of Medical Sciences, Shanghai Jiaotong University School of Medicine, Shanghai 200025, China; 5 Institute of Molecular Immunology, School of Biotechnology, Southern Medical University, Guangzhou 510515, China; 6 School of Life Sciences, Fudan University, Shanghai 200433, China

**Keywords:** apoptotic cell-in-cell death, emperitosis, immune cytotoxic cells, granzyme B, vacuole formation

## Abstract

A cell-in-cell process refers to the invasion of one living cell into another homotypic or heterotypic cell. Different from non-apoptotic death processes of internalized cells termed entosis or cannibalism, we previously reported an apoptotic cell-in-cell death occurring during heterotypic cell-in-cell formation. In this study, we further demonstrated that the apoptotic cell-in-cell death occurred only in internalized immune killer cells expressing granzyme B (GzmB). Vacuole wrapping around the internalized cells inside the target cells was the common hallmark during the early stage of all cell-in-cell processes, which resulted in the accumulation of reactive oxygen species and subsequent mitochondrial injury of encapsulated killer or non-cytotoxic immune cells. However, internalized killer cells mediated rapid bubbling of the vacuoles with the subsequent degranulation of GzmB inside the vacuole of the target cells and underwent the reuptake of GzmB by killer cells themselves. The confinement of GzmB inside the vacuole surpassed the lysosome-mediated cell death occurring in heterotypic or homotypic entosis processes, resulting in a GzmB-triggered caspase-dependent apoptotic cell-in-cell death of internalized killer cells. On the contrary, internalized killer cells from GzmB-deficient mice underwent a typical non-apoptotic entotic cell-in-cell death similar to that of non-cytotoxic immune cells or tumor cells. Our results thus demonstrated the critical involvement of immune cells with cytotoxic property in apoptotic cell-in-cell death, which we termed as emperitosis taken from emperipolesis and apoptosis. Whereas entosis or cannibalism may serve as a feed-on mechanism to exacerbate and nourish tumor cells, emperitosis of immune killer cells inside tumor cells may serve as an in-cell danger sensation model to prevent the killing of target cells from inside, implying a unique mechanism for tumor cells to escape from immune surveillance.

Cell-in-cell refers to one or more living cells (referred as effector cell) initiatively entering into another cell (referred as target cell) to form cell-in-cell structures, an old biological phenomenon that can be traced back one hundred years ago but has been long-term overlooked.^1^ Until recent, cell-in-cell has attracted more attention, not only because of the elaborate investigations on its characteristics and mechanisms^2, 3, 4, 5, 6^ but also because of its close relationship with clinical pathogenesis.^7, 8, 9, 10^

Cell-in-cell occurs *in vitro* or *in vivo* either homotypically or heterotypically representing a unique intercellular interactions of diverse cells.^11^ Most of the homotypic cell-in-cell structures occur between sibling tumor cells, whereas heterotypic cell-in-cell structures are formed between immune cells and tumor or other various tissue cells, which was previously termed as ‘emperipolesis'.^12^ Internalized effector cells can either undergo mitosis inside or be released intactly from the target cells. However, majority of them succumb to cell-in-cell death.^13^

So far, three kinds of cell-in-cell death have been reported with shared and distinct characteristics, including cannibalism, entosis and apoptotic cell-in-cell death.^4, 5, 6^ Cannibalism is described to be a process that metastatic tumor cells under starvation exhibit the ability to actively take or ‘eat' other homotypic or heterotypic live or dead cells, which is similar to phagocytosis.^6, 7^ Degradation of effector cells inside cannibalistic cells relies on the acidic digestive machinery in caveosomes that requires scaffolding proteins like caveolin-1 or ezrin as well as the activation of proteolytic enzymes. This lysosome-dependent cannibalistic cell-in-cell death mediates the subsequent nutrient supplement under starvation. Alternatively, this process reflects one of the mechanisms of tumor cells to escape from immune attack.^6, 14, 15^ Entosis is defined as the homotypic invasion of tumor or epithelial cells into their neighboring cells, triggered by extracellular matrix detachment. Internalized cells are trapped in the vacuole of the target cells (entotic vacuole). Autophagy proteins from the target cell, such as ATG5, ATG7 and the class III PI3-kinase VPS34, mediate the fusion of lysosomes from target cells with entotic vacuoles, which is marked by a proceeding transient recruitment of microtubule-associated protein 1A/1B-light chain 3 (LC3) to entotic vacuoles and followed by a unique autophagosome-independent lysosomal death of the internalized cells.^3^ It is suggested that entosis serves as a homeostatic mechanism to inhibit metastasis through internalizing effector cells. In addition, entosis may also contribute to tumor progression through the induction of aneuploidy.^2^

It has been generally accepted that penetration of lymphocytes through tumor cells represents a special form of immune attack, a so-called ‘Trojan horse' effect.^16, 17, 18^ However, our early and recent studies as well as those from others provide evidence that cell-in-cell death is the major destination of internalized immune cells characterized as caspase-dependent apoptotic cell-in-cell death, a process different from cannibalism or entosis.^4, 16, 18^ The mechanisms of the apoptotic cell-in-cell death occurring between heterotypic cell-cell interaction and its discrepancy with cannibalism and entosis are still far from conclusive.

Here, by expanding the spectrum of cell lines including either immune cell lines or freshly isolated human and mouse lymphocytes, we revealed that not all of immune cells underwent apoptotic cell-in-cell death. Only those with cytotoxic activities (killer cells) exerted the behavior of apoptotic cell-in-cell death when invading into tumor cells. In contrast, the internalized immune cells without cytotoxic activities manifested entotic cell-in-cell death. On the basis of these observations, we further elucidated the mechanisms underlying apoptotic cell-in-cell death of immune killer cells inside tumor cells as well as discussed its implicated clinical significance.

## Results

### Emperitosis, an apoptotic cell-in-cell death process, occurs in heterotypic immune killer cells inside tumor cells

**Figure 1:**
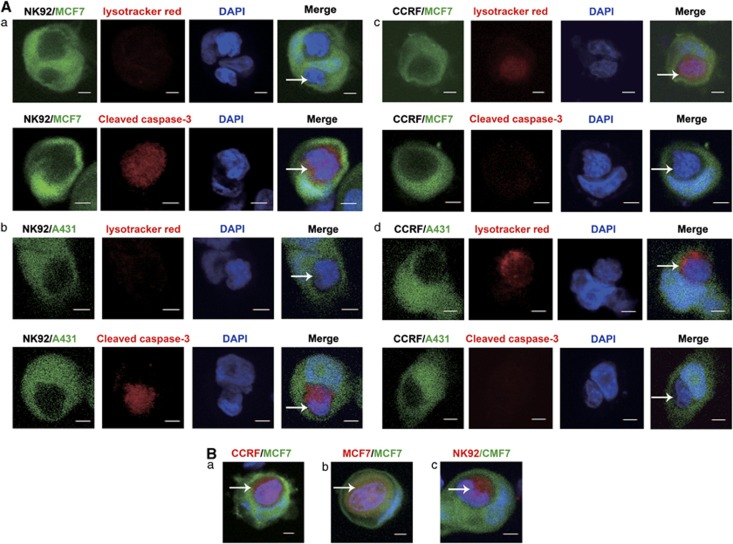
Comparative characterization of cell-in-cell death. (**A**) Cell-in-cell death of NK92 (killer cells) or CCRF (non-cytotoxic cells) underwent different pathways. Caspase-3 was activated in cytotoxic NK92 cells inside target cells MCF7 (a) and A431 (b) rather than lysosome activation. Differently, non-cytotoxic CCRF displayed lysosome activation in MCF7 (c) and A431 (d) cells. Arrows indicated internalized effector cells. Scale bar: 5 *μ*m. (**B**) Nucleus morphology was compared among homotypic entosis, heterotypic entosis or emperitosis. The nucleus of internalized CCRF and MCF7 cells was not condensed in CCRF/MCF7 (a) or MCF7/MCF7 (b) cell-in-cell structures (observed at 12 h), when lysosome were activated (red) in the effector cells. However, caspase-3-activated NK (red) cell was karyopyknosis in NK92/MCF7 cell-in-cell structure (observed at 8 h) (c). Arrows indicated the nucleus of internalized cells. Scale bar: 5 *μ*m

According to our previous study on the investigation of cell-in-cell structure formation either homotypically or heterotypically by using more than 20 tumor cell lines as target cells and more than 10 types of immune cells as effector cells,^13^ we once supposed that apoptotic cell-in-cell death exclusively occurred during heterotypic cell-in-cell structure formation. However, when extending the spectrum of immune cells as effector cells, we found that internalized immune cells under investigation died in two manners, either lysosomal entosis or apoptotic cell-in-cell death. Consistent with previous study, caspase-3 activation in internalized NK92 cells occurred within 6 h coculture in either MCF7 (Figure 1Aa) or A431 (Figure 1Ab) target cells. Unexpectedly, the death of CCRF cells manifested a feature of entosis, exhibiting lysotracker positive and cleaved caspase-3-negative patterns following cell-in-cell formation with MCF7 (Figure 1Ac) or A431 (Figure 1Ad), which was similar to homotypic cell-in-cell death of MCF7.^4, 5^ Examining closely, chromosomes in CCRF remained loose before degradation and nucleic volume did not alter inside MCF7 (Figure 1Ba), the same as observed in homotypic MCF7 cell-in-cell structure (Figure 1Bb). On the contrary, internalized NK92 cells in MCF7 were characterized as apoptosis with remarkable cytoplasmic and nucleus condensation (Figure 1Bc). Summarizing our investigation (Table 1), we found that RAJI cells as well as primary B cells and monocytes freshly isolated from human peripheral blood mononuclear cells (PBMCs) underwent entosis, whereas freshly isolated CD4^+^, CD8^+^T cell, CD56^+^NK cells and *in vitro*-generated cytokine-induced killer (CIK) or lymphokine-activated killer (LAK) cells underwent apoptotic cell-in-cell death in MCF7 target cells. Considering the common target cells investigated in either homotypic or heterotypic cell-in-cell structure formation, our results suggested that the manner of cell-in-cell death was dependent on the type of the effector cells rather than the target cells.

Comparing the property of the effector cells in Table 1, we found that most of the immune cells undergoing apoptotic cell-in-cell death possessed the cytotoxicity capacity, such as CD8^+^T cells, NK cells (both primary cells and cell lines), CIK cells and LAK cells, whereas cells undergoing entosis exhibited less cytotoxicity such as B cells or monocytes. These results strongly implied that apoptotic cell-in-cell death might be closely associated with the cytotoxic property of immune cells. Immune cells with cytotoxicity (referred as killer cells) were inclined to undergo apoptotic cell-in-cell death. To better distinguish apoptotic cell-in-cell death from lysosomal entosis or cannibalism, we termed the apoptotic cell-in-cell death as emperitosis (taken from emperipolesis and apoptosis).

### Immune cells with cytotoxicity undergo emperitosis in a GzmB-dependent manner

**Figure 2:**
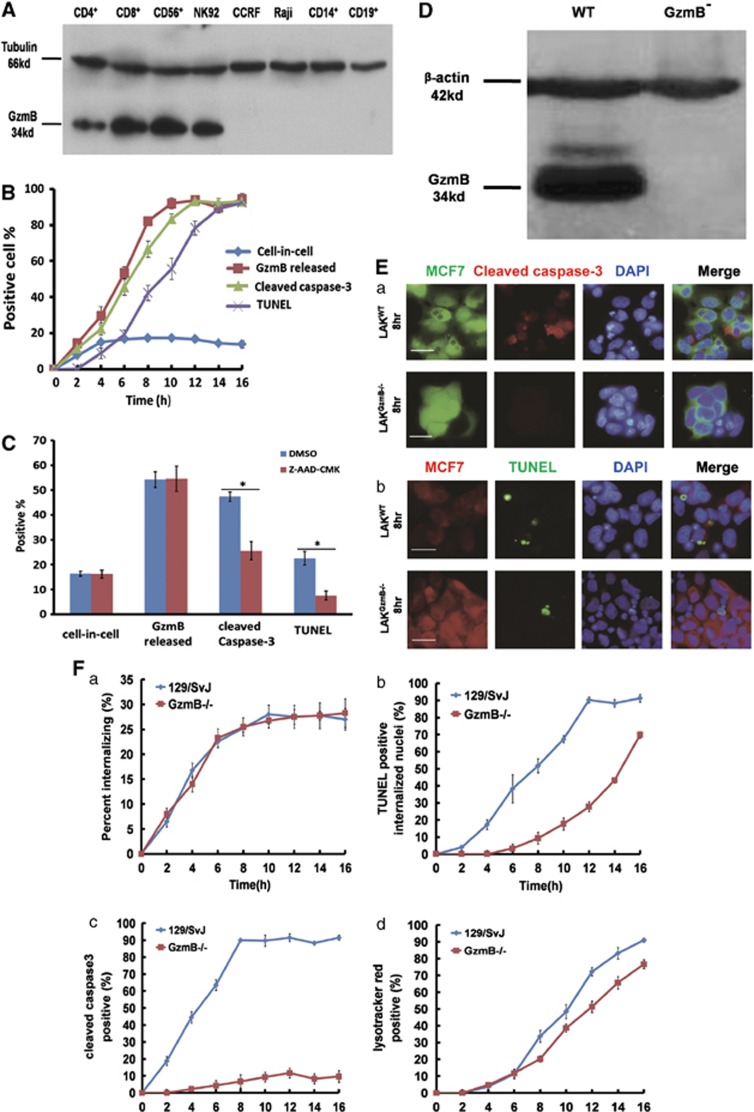
GzmB released by killer cells induced apoptotic cell-in-cell death. (**A**) Expression of GzmB in cytotoxic immune cells (CD4^+^, CD8^+^, CD56^+^ and NK92) or non-cytotoxic immune cells (CCRF, RAJI, CD14^+^ and CD19^+^) by western blot analysis. (**B**) In parallel analysis of internalizing cells (blue curve), GzmB release (red curve), caspase-3 activation (green curve) or TUNEL assays (purple curve) of internalized cytotoxic immune cells at different time points during NK92/MCF7 cell-in-cell formation. (**C**) Quantification of internalized cells, GzmB release, cleaved caspase-3 and TUNEL assays of NK92 cells with (red bars) or without (blue bars) Z-AAD-CMK treatment at 6 h of cell-in-cell formation. (**D**) Determination of GzmB expression in LAK cells from wild-type or *GzmB*^*−/−*^129/SvJ mouse by western blot analysis. (**E**) (a) Comparison of caspase-3 activation (red) in LAK cells from either wild-type (upper panel) or *GzmB*-deficient mice (lower panel) when internalized in MCF7 target cells (CellTracker green). (b) LAK cells from both wild-type (upper panel) and *GzmB*^*−/−*^ mouse (lower panel) internalized in MCF7 cells (CellTracker red) were positive for TUNEL at 8 h. Scale bar: 20 *μ*m. (**F**) Kinetic quantification of internalized cells (a), TUNEL (b), cleaved caspase-3 (c) and lysosomal activation (d) in wild-type (blue curves) or *GzmB*^*−/−*^ mouse LAK cells (red curves). One representative experiment of three independent experiments was shown. Data were means±S.D. *P<0.05

It is well known that cytotoxic cells attack the target cells through secreting granzymes (especially granzyme B, GzmB) by cytoplasmic degranulation. The released GzmB enters the target cells and cleaves caspases which in turn activates caspase-dependent DNase, induces the fragmentation of DNA and apoptosis of the target cells.^19, 20, 21^ To determine the involvement of GzmB in apoptotic cell-in-cell death of immune killer cells, GzmB levels were first analyzed in a panel of immune cells investigated. Western blot results indicated abundant GzmB expression in cytotoxic cells, even in CD4^+^T cells which also underwent apoptotic cell-in-cell death inside tumor cells. Very little GzmB was detectable in non-cytotoxic immune cells (Figure 2A). Moreover, killer cells such as NK92 released GzmB with the same dynamics as the occurrence of apoptotic cell-in-cell death through monitoring caspase-3 activation and cell-in-cell death simultaneously (Figure 2B). Interference of GzmB activity by Z-AAD-CMK, a GzmB-specific irreversible inhibitor, did not affect cell-in-cell structure formation and GzmB release but significantly inhibited caspase-3 activation and apoptotic cell-in-cell death of killer cells (Figure 2C). Furthermore, mouse LAK cells deficient in *GzmB* (Figure 2D) showed less and delayed cell-in-cell death (Figures 2Eb and 2Fb) featured with normal cell-in-cell structure formation (Figure 2Fa) and the absence of cleaved caspase-3 (Figures 2Ea and 2Fc). These results strongly supported that GzmB released by killer cells had crucial roles in apoptotic cell-in-cell death of immune killer cells.

### Trapping within the vacuole of the target cells and re-endocytosis of GzmB leads to apoptotic cell-in-cell death of internalized killer cells

**Figure 3:**
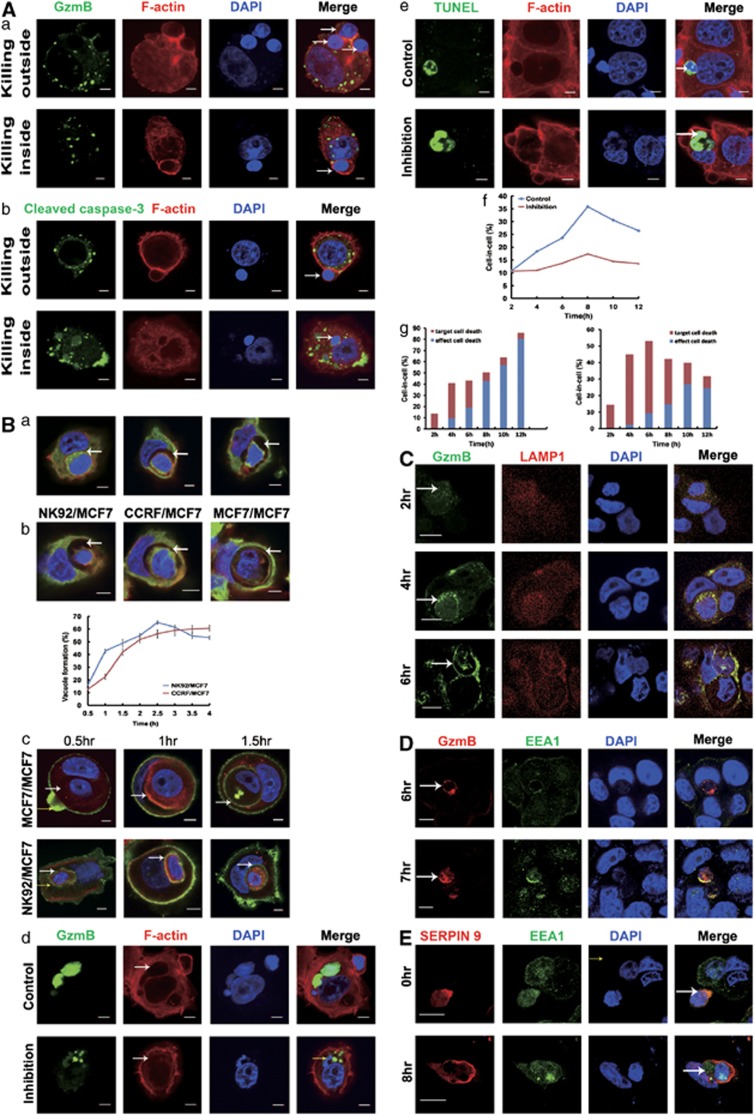
Apoptotic cell-in-cell death induced by re-endocytosis of GzmB from the vacuole structure. (**A**) (a) GzmB released to the cytoplasm of the target cells by NK92 cells from either outside (upper panel) or inside (lower panel) of MCF7 cells. (b) Caspase-3 activation in NK92 cells (upper panel) or target cells (lower panel). White arrow indicated NK92. Scale bar: 5 *μ*m. (**B**) (a) Successive changes of vacuolation in MCF7 from wrapping (left) to ‘trench' (right) in cell-in-cell structure formation between NK92 and MCF7 cells. F-actin (red), tubulin (green) and nuclei (DAPI, blue) were labeled separately. White arrows indicate vacuolation. (b) Vacuole formation in cell-in-cell structures. Vacuoles containing NK92 (left panel), CCRF (middle panel) or MCF7 (right panel) cells were formed inside MCF7 cells (upper panel). Kinetics of vacuole formation in cell-in-cell structures formed by NK92/MCF7 (blue curve) or CCRF/MCF7 (red curve) (lower panel). Data are means±S.D. from one representative experiment measured in triplicate. Scale bar: 5 *μ*m. (c) Ezrin clustering during vacuole formation. When one MCF7 cell penetrated into another MCF7 cell undergoing entosis (upper panel), ezrin (Green) was accumulated at the membrane of vacuole inside MCF7 for 4 h (left panel). In emperitosis (lower panel), when NK92 effector cells invaded into the MCF7 target cell, ezrin accumulation surrounding the vacuole has shorten to 2 h (left panel). (d) GzmB distribution in MCF7 target cells with or without treatment of cytochalasin B and nocodazole. NK92 cell was enveloped by vacuole (white arrow, upper panel), and GzmB was diffused in the vacuole of target cell (yellow arrow, upper panel). Treatment with cytochalasin B and nocodazole led to the loss of the vacuolar structure integrity (white arrow, lower panel) and the release of GzmB into the cytoplasms of the target cells (yellow arrow, lower panel). (e) Immunofluorescent staining and TUNEL assays showed that internalized effector cell (arrow) was positive for TUNEL (green) without inhibitor treatment (upper panel), whereas target cell (arrow) was positive for TUNEL following cytochalasin B (10 *μ*M) and Nocodazole (10 *μ*M) treatments (lower panel). Inhibitor treatment thus decreased the ratio of cell-in-cell structures (f) and reversed the cell death proportion of target cells to effector cells in cell-in-cell structures (g, left: without inhibitor; right: with inhibitor). (**C**) Gradual degranulation and gathering of GzmB in the vacuole around internalized NK92 cells through colocalization of GzmB (green) and LAMP1 (red). MCF7 target cells were negative for GzmB even at 6 h post cell-cell incubation. Arrows indicated GzmB. Scale bar: 10 *μ*m. (**D**) GzmB re-entered NK92 cells through endocytosis. GzmB gathered surrounding NK92 cells without the formation of endosomes after 6 h of cell-in-cell structure formation and was not wrapped by the endosomes of NK92 cells (upper panel) until 7 h (bottom panel), demonstrated by colocalization of GzmB (red) and EEA1 (green) within the target cells. Arrows indicated gathering GzmB. Scale bar: 10 *μ*m. (**E**) Immunofluorescent staining showed that the scope of serine proteinase inhibitor SERPIN 9 (red) was far transcended by that of re-endocytosed GzmB (green) in internalized NK92 cells at 8 h, indicating the failure to inhibit GzmB activity. Arrows indicated GzmB distribution. Scale bar: 10 *μ*m

It has been reported that in some situations killer cells can endocytose activated GzmB to initiate self-apoptosis in order to maintain endogenous homeostasis.^22, 23, 24^ In our study, the observation that GzmB released by internalized killer cells initiated a suicide process rather than attacked the target cells, to some extent, resembles the ‘in-cell' suicide behavior of killer cells. Actually, when internalized killer cells were not encapsulated intactly by the vacuolar structure of the target cells or at the very early stage of vacuole formation, they released GzmB directly into the cytoplasm of the target cells. This led to the apoptosis of the target cells that was similar to the killing of the target cells by GzmB from outside (Figure 3A) in accordance with previous observations.^16, 18^ With the formation of the vacuoles inside MCF7 target cells, the vacuole wrapping the NK92 cells expanded rapidly, forming a ‘trench' between two cells (Figure 3Ba, from left to right). When we observed the vacuole formation between heterotypic NK92/MCF7, CCRF/MCF7 and homotypic MCF7/MCF7 interactions, all three types of vacuoles were apparent with the entity of vacuole membrane around internalized effector cells (Figure 3Bb, upper panel, white arrow). However, when we further compared the kinetics of vacuole formation between entosis and emperitosis in heterotypic cell-cell interactions, vacuole formation following killer cell invasion was relatively earlier in emperitosis than that of heterotypic entosis (Figure 3Bb, lower panel). As ezrin is demonstrated to play critical roles during heterotypic cell-in-cell structure and vacuole formation,^4^ we further explored the kinetic engagement of ezrin as another indicator to compare the vacuole formation in entosis or emperitosis. When we tracked the ezrin clustering at the early stages of two processes in parallel, we found that ezrin accumulation around the vacuole was more rapid during emperitosis (Figure 3Bc, lower panel) than entosis (Figure 3Bc, upper panel) (green labeling, white arrow indicated). Ezrin clustering at the interface of vacuole was earlier in emperitosis than in entosis, which was consistent with the results from the vacuole formation.

When NK92 and MCF7 cocultured with the presence of cytochalasin B and nocodazole for 2 h, which functioned to break the microfilament and interrupt the bubbling of vacuoles in the target cells, MCF7 cells suffered apoptosis (Figure 3Be). This was largely due to the fact that the vacuoles failed to encapsulate the internalized NK92 cells completely and degranulated GzmB has leaked into the cytoplasm of the target cells (Figure 3Bd). With the decreased cell-in-cell formation (Figure 3Bf), a significant increase in the percentage of target cell death and a decrease in that of killer cell death was observed (Figure 3Bg, right). On the contrary, the death ratio of target cell to internalized killer cell was remarkably reversed without the treatment of these two inhibitors (Figure 3Bg, left). These further demonstrated that the vacuole formation of the target cells facilitated the trapping of released GzmB inside the vacuole and prevented the killing of the target cells from inside by internalized killer cells.

GzmB was restrained in the granules of internalized NK92 at the early time point (2 h), as GzmB was colocalized with lysosomal-associated membrane protein 1 (LAMP1) in internalized NK92 cells after entering MCF7 tumor cells (Figure 3C, upper row). Four hours later, abundant GzmB became trapped in the vacuoles of cell-in-cell structures, indicating the failure of NK92 cells to release GzmB into the cytoplasm of the MCF7 target cells (Figure 3C, middle row). At 6 h, GzmB was limitedly distributed in the cytoplasm of internalized NK92 cells as well as in the ‘trench' around NK92 cells without overlapping of LAMP1, indicating the restricted distribution of GzmB inside the vacuole of MCF7 target cells (Figure 3C, lower row).^21, 24^ Therefore, another interesting question arose as how restrained GzmB inclined to induce self-apoptosis of the internalized cells, while keeping the target cells alive. It has been revealed that degranulated granzymes could cross cell membrane through the endo-lysosomal pathways and subsequently release into the cytoplasm to induce apoptosis.^25, 26^ We further examined the expression of early endosome antigen 1 (EEA1), an endocytic vacuole marker, on the membrane of internalized NK92 cells. The distribution of GamB during cell-in-cell formation was coincident with endocytosis process. As shown in Figure 3D, GzmB was re-endocytosed back into the cytoplasm of internalized NK92 cells through endosomes (7 h). It is already reported that cytotoxic immune cells express serine protease inhibitors for protection from auto-secreted cytotoxic molecules.^27, 28^ As expected, GzmB and serine proteinase inhibitor 9 (SERPIN 9) were colocalized in the very beginning of cell-in-cell structure formation (Figure 3E, upper row). With the scattering of degranulated GzmB over the cytoplasm of internalized NK92 cells, the segregation of GzmB and SERPIN 9 was observed with the apoptosis of NK92 cells (Figure 3E, lower row).

Taking all these strands of evidence together, we supposed that during killer-tumor cells interaction, rapid vacuole formation by tumor cells may obstruct the introduction of GzmB into the cytoplasm of the target cells, therefore more favorable to initiate a re-endocytosis of the activated GzmB back into the internalized killer cells and cause an ‘in-cell' apoptotic suicide of the killer cells.

### Heterotypic entosis and apoptotic cell-in-cell death display common events in the early phase of cell-in-cell structure formation

**Figure 4:**
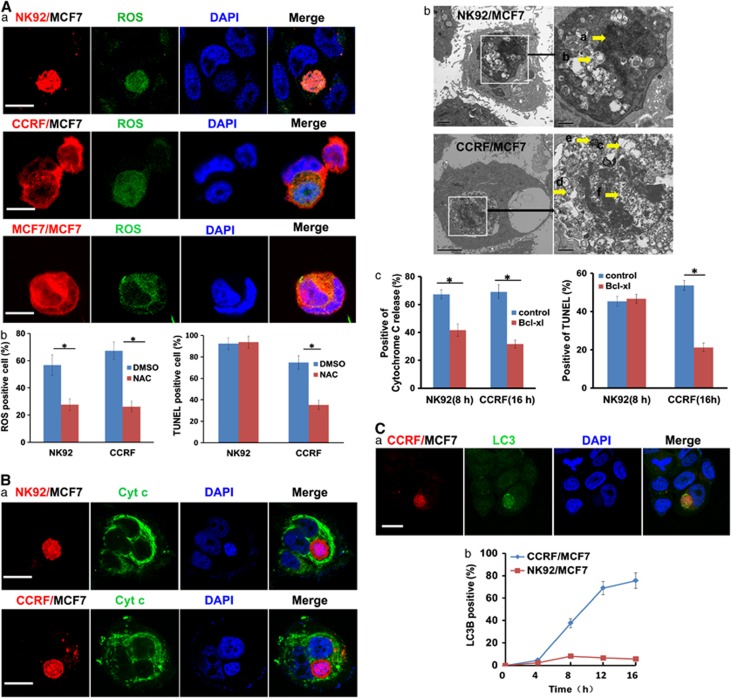
Mechanism investigation on the early phase of heterotypic entosis and emperitosis. (**A**) ROS accumulation in cell-in-cell structure formation. (a) Accumulation of ROS (green) in NK92 killer cells (CellTracker red; top panel), non-cytotoxic CCRF cells (CellTracker red; middle panel) or MCF7 cells (CellTracker red; bottom panel), respectively. Scale bar: 10 *μ*m. (b) Quantification of ROS accumulation and TUNEL-positive internalized effector cells with or without the treatment of ROS inhibitor NAC. (**B**) Cyt c release in cell-in-cell structure formation. (a) Large amount of Cyt c (green) was released by both NK92 cells (CellTracker red; upper panel) and CCRF cells (CellTracker red; lower panel). Green color indicated the release of Cyt c from the mitochondria by internalized cells. Scale bar: 10 *μ*m. (b) Under TEM observation, an NK92 cell in the cytoplasm of target cell MCF7 (upper panel) exhibited karyopyknosis (arrow a) and mitochondrial swelling (arrow b), whereas the internalized CCRF cell (lower panel) showed up a shade of swollen mitochondrial (arrow c), the presence of autophagosomes (arrow d) and autophagolysosome (arrow e) and chromosomal degradation (arrow f). (c) Quantification of Cyt c release and TUNEL-positive internalized cells in control and Bcl-xL-treated cytotoxic NK92 cells or non-cytotoxic CCRF cells, *P<0.05. (**C**) Autophages in cell-in-cell structure formation. (a) Accumulation of autophagic bodies in internalized CCRF cells (CellTracker red) by LC3B (green) labeling. Scale bar: 10 *μ*m. (b) LC3 expression was increased in CCRF cells (blue curve) but not in NK92 cells (red curve)

Encirclement of the internalized effector cells through vacuole formation inside the target cells is demonstrated to be the key step to initiate entosis after homotypic cell-in-cell structure formation. The inner environment of the vacuoles causes hypoxia and initiates the autophagy and heterophagy, finally leading to a lysosome-dependent entotic pathway of the invading cells.^3^ As we also observed the occurrence of entosis between heterotypic immune-tumor cell interactions, we further questioned whether it was the same situation in heterotypic entosis in non-cytotoxic immune-tumor cell-in-cell structures as in homotypic tumor-tumor cell-in-cell structures. In the early stage of cell-in-cell formation, the intracellular accumulation of reactive oxygen species (ROS) was comparable in NK92-MCF7 (Figure 4Aa, upper panel) and CCRF-MCF7 cell-in-cell structures (Figure 4Aa, middle panel) as well as in MCF7 homotypic cell-in-cell structures (Figure 4Aa, lower panel). However, inhibition of ROS accumulation with N-acetyl-L-cysteine (NAC) dramatically reduced heterotypic entosis but not emperitosis (Figure 4Ab). Intracellular ROS accumulation results in mitochondrial injury, which is a critical sensor to initiate the mitochondrial apoptotic pathway.^29, 30^ As expected, cytochrome c (Cyt c) release (Figure 4Ba) and mitochondrial swelling (Figure 4Bb) were observed in both non-cytotoxic immune cells and killer cells invading tumor cells. Consistent with the results from ROS accumulation inhibition, addition of Bcl-xL led to the impairment of Cyt c release in both heterotypic entosis and emperitosis (Figure 4Bc, left). However, cell-in-cell death only decreased in heterotypic CCRF-engaged entosis not in NK92-engaged emperitosis (Figure 4Bc, right). We also observed the accumulation of LC3B in internalized cells during entosis of CCRF cells inside MCF7 (Figure 4Ca) but rarely in emperitosis of NK92 in MCF7 (Figure 4Cb). These results supported that vacuole formation around invading cells was a common step during the early stages of entosis and emperitosis regardless of the effector cell types, but different in vacuole bubbling afterward.

## Discussion

Cell-in-cell represents a unique form of cell-cell interactions affecting both effector cells and target cells. With the research progresses on cell-in-cell death of the internalized effector cells, three similar but different cell-in-cell death processes are introduced in the literatures. They are entosis, cannibalism and apoptotic cell-in-cell death.^11, 31^ The mechanisms of entosis and cannibalism have been clearly illustrated. In entosis, the target cells wrap the invading cells with a cytoplasmic membrane to create a starvation environment (entotic vacuole). Through the recruitment of heterophagic molecules such as LC3 on the vacuole membrane, lysosomes of target cells are fused with effector cell containing entotic vacuoles and a lysosomal pathway is initiated to degrade the invading cells inside the vacuoles.^3^ Unlike phagocytosis with dead cells^32^ or entosis of live cells, cannibalism has provided more extensive selection for internalized cells, either live or dead sibling cells or lymphocytes into target cells.^6^ Covaculae-1 has a leading role in that it induces the lysosomes gathering on to the target cell vacuoles around internalized cells.^15^ We previously reported the existence of apoptotic cell-in-cell death and speculated it as a unique characteristic of heterotypic cell-in-cell structures, particularly of the immune-tumor cell-in-cell structures. However, when we extended the effector cell lineages for investigation, we found that whether cell lines or freshly isolated human peripheral blood immune cells underwent typical entosis or apoptotic cell-in-cell death inside tumor cells depended on the types of the invading cells (Table 1). Only cytotoxic immune cells (killer cells) were inclined to undergo apoptotic cell-in-cell death, in which GamB from internalized killer cells was involved in the apoptotic death pathway. Results from *GzmB*-knockout mouse supported the critical roles of GzmB in inducing apoptotic cell-in-cell death of killer cells (Figure 2F).

Immune killer cells such as NK cells or cytotoxic CD8^+^ T cells possess the properties to secrete certain molecules such as GzmB or perforin to orchestrate the cytotoxicity toward the target cells.^19, 20, 21^ It has been reported that under certain circumstances, NK cells also initiated GzmB-mediated suicide, which is considered to be an intrinsic homeostasis mechanism.^22, 23, 24^ The involvement of GzmB in apoptotic cell-in-cell death of killer cells inside the target cells recapitulates the suicide process from outside, a process called the ‘killing from inside target cells' or ‘Trojan horse effect' (Figure 3).^16^ However, it is curious that GzmB released by killer cells inside the target cells does not attack the target cells. Our results demonstrated that the rapid vacuole formation by the target cells trapped the GzmB inside the vacuoles rather than released it in the cytoplasm of the target cells, which in turn protected the target cells from GzmB attack. The accumulated GzmB was retaken up and transferred quickly back into the cytoplasm of invading killer cells through the mediation of endosomes marked by EEA1 on the membrane of these cells and triggered a GzmB-engaged apoptotic cell-in-cell death of killer cells inside the target cells.

In fact, entosis and emperitosis share the common step of vacuole formation as well as ROS accumulation in the early stage of cell-in-cell structure formation.^5^ However, with the degranulation of GzmB by killer cells inside vacuoles this finally facilitates the in-cell apoptotic suicide of killer cells different from the entosis of non-immune killer cells inside. In our study, we compared the kinetics of vacuole formation between entosis and emperitosis and found that the vacuole formation during emperitosis was faster than that of entosis (either homotypic or heterotypic). When the vacuole formation was impaired, there displayed cell death of the target cells with the diffuse of GzmB in the cytosol of the target cells. Therefore, rapid vacuole formation and the subsequent formation of ‘trench' in the vacuoles guaranteed the restriction of GzmB in the vacuoles, leading to the reuptake of degranulated GzmB by the killer cells themselves. How non-cytotoxic or cytotoxic immune cells provide different signals to determine the rate of vacuole formation will become future research focus, which might provide new strategies for target screening in multiple diseases.

Furthermore, the accumulation of ROS was observed in the early stages of hetero-entosis and emperitosis which is similar to homotypic entosis.^3, 5^ However, inhibiting ROS generation and Cyt c release significantly suppressed the hetero-entosis but did not stop emperitosis. The LC3B aggregation was only observed in the early stage of emperitosis at a very low level and reduced quickly with the development of cell-in-cell death (Figure 4Cb). These results suggest that either entosis or emperitosis shares certain features during the early invasion stage, whereas the danger signals delivered by different types of invading cells for the vacuolation inside the target cells are in divergence afterwards, which finally results in the different cell-in-cell death pathways. In fact, when treated entotic cells with lysosome inhibitor Con A in homotypic entosis, there happened a transformation of lysosomal cell-in-cell death into a caspase-dependent apoptosis^5^ (and our own unpublished data).

More significantly, during the pathogenesis such as inflammation or carcinoma, most of the immune cells infiltrating into local regions are those activated or with cytotoxicity.^33^ In fact, it does not absolutely benefit from the infiltration of these lymphocytes for the amelioration of the pathology. The infiltration of killer cells in local might fasten the elimination of these cells through cell-in-cell structure formation according to our study. After activating the immune cells *in vitro*, we observed that the activation of the immune cells enhanced the tendency of heterotypic cell-in-cell formation. When adding the supernatant of toxin-treated PBMCs in the cell-in-cell formation, the frequency of cell-in-cell formation dramatically increased. In addition, the impact of starvation was more dramatic on entosis than on emperitosis when we used low serum concentration to mimic this condition (data not shown). To our prediction, the presence and status of killer cells and their interactions with tissue cells are more likely to deliver an ‘in-cell danger signal' when entering the target cells, whose destination is to eliminate the invading killer cells by apoptosis inside with the abundance of GzmB. Accordingly, emperitosis might be the alternative form of entosis with different aims of cellular biological behaviors. To elucidate the exact biological significance will facilitate our understanding of how cell-in-cell initiates the ‘in-cell danger' recognition for the most efficient self-protection depending on the type of internalized cells.

## Materials and Methods

### Cell culture

A431, HCT8, MCF7 and HepG2 cells were maintained routinely in DMEM complete medium with 10% FBS (Hyclone, Logan, UT, USA) and 100 *μ*g/ml penicillin–streptomycin (Invitrogen, Carlsbad, CA, USA). NK92 cell line was gifted by Dr. Hai-ming Wei (University of Science and Technology of China, Hefei, China) and maintained in MEM-alpha medium with 15% fetal bovine serum (FBS) (Invitrogen) supplemented with 100 *μ*g/ml penicillin–streptomycin (Invitrogen). CIK and murine LAK cells were prepared as reported previously using cytokines rhIFN-*γ*, rhIL-1*α* and rhIL-2 (R&D Systems, Minneapolis, MN, USA).^34^ Cells were cultured at 37 °C with 5% CO_2_.

### Animals

*Gzmb*^−/−^mice (*GzmB*^*−/−*^*/PGK-neo*) and wild-type littermates (129/SvJ)^35^ were generously provided by Dr. Yu-fang Shi (Institute of Health Science, SIBS, CAS, Shangai, China). Animals were matched for age and gender in each experiment. All animal experiments were approved by the Institutional Animal Care and Use Committee.

### Magnetic-activated cell sorting

PBMCs were isolated by Ficoll-hypaque gradient centrifugation using Leukapheresis (Pharmacia Fine Chemicals, Rahway, NJ, USA). Primary CD4^+^T, CD8^+^ T, CD56^+^ NK, CD19^+^ B cells and CD14^+^ monocytes were positively sorted by magnetic beads according to the manufacturer's instructions (Miltenyi Biotec, Bergisch Gladbach, Germany). The purity was determined by flow cytometry.

### *In vitro* cell internalization assays

Target tumor cell suspension was stained with 2.5 *μ*M CellTracker Green dye (Invitrogen, San Diego, CA, USA) for 30 min at 37 ^o^C in the absence of serum and incubated for 4 h with equivalent number of immune cells prestained with 2.5 *μ*M CellTracker Red dye (Invitrogen). The mixture of target and immune cells was cytospun onto the slides in a Cytocentrifuge 7620 (Wescor, Logan, UT, USA) at 500 r.p.m. for 3 min. DNA was stained with DAPI (Sigma-Aldrich, St. Louis, MO, USA) for determination of internalization. Coverslips were supported on slides by grease pencil markings and mounted in Vectashield (Vector Laboratories, Burlingame, CA, USA). Cell-in-cell structures were examined under a laser-scanning confocal microscope scan head LSM510 NLO (Carl Zeiss, Göttingen, Germany) mounted transversely to the Axiovert 200 inverted microscope (Carl Zeiss) with a 40 × 1.3 numerical aperture PlanApo objective. Optical section series were collected with a spacing of 0.4 *μ*m in the z axis through 0.12 *μ*m thickness of cell-in-cell complex. Digital data were exported into Adobe Photoshop for image preparation. The percentages of cell-in-cell structures were calculated by counting 400 target cells with a double-blind method.

Time-lapse microscopy was performed as described previously.^5^ Briefly, pDsRed-expressing tumor cells labeled with Hoechst 33342 (Invitrogen) were adherent on 35-mm glass bottom cell culture dishes (Nest Biotechnology, Jiangsu, China). Killer cells stained with Hoechst or Celltracker green were added. Fluorescence and differential interference contrast images were obtained every 1 min and the results were represented for the indicated time courses.

In some experiments, 10 *μ*M Bcl-xL protein (Calbiochem, San Diego, CA, USA) was added in the *in vitro* culture. Antibodies against F-actin (Invitrogen, Molecular Probes, Eugene, OR, USA), LAMP1 (Abcam, Cambridge, MA, USA), EEA1 (Abcam), SERPIN 9 (Abcam), cleaved caspase-3 (Millipore, Temecula, CA, USA), human GzmB (Millipore) and LC3B (Millipore) and Cytochrome c (Millipore) were used for further labeling.

### Detection of vacuole formation

For vacuolation assay, tumor cells were seeded on coverslips and incubated with effector cells. AlexaFluor 594-phalloidin (Invitrogen) and anti-tubulin antibody (Abcam) were used to measure the distance between the vacuole and internalized cell-in-cell structures every 30 min with FV1000 software (Olympus, Tokyo, Japan). We defined ‘trench' as when the distance between vacuole and internalized cell was more than 2.5 *μ*m. The proportion of trench among vacuolation was calculated by random selection of 10 different fields. In some experiments, cells were incubated in the presence of 10 *μ*M cytochalasin B and 10 *μ*M nocodazole (Sigma-Aldrich) for the indicated time to determine the role of microfilament and the bubbling of the vacuole in the target cells during vacuole formation.

### Detection of ezrin clustering

For the tracking of ezrin accumulation during cell-in-cell, tumor cells were seeded on coverslips and incubated with effector cells. Fix cells with 4% paraformaldehyde (Sigma-Aldrich) every 15 min and permeabilize them using 0.1% Triton X-100. Cells were incubated with anti-ezrin antibody (Abcam) for 60 min. After washing with PBS for three times, the cells were incubated with FITC-conjugated rabbit anti-mouse IgG at room temperature for 1 h. AlexaFluor 594-phalloidin and DAPI dye (Invitrogen) were incubated with cells for the detection of F-actin and the nuclei, respectively. The slides were observed under laser-scanning confocal microscope (Carl Zeiss).

### Detection of cell-in-cell death

TUNEL assay was performed to determine cell-in-cell death according to the manufacturer's protocol (Promega, Madison, WI, USA). Briefly, cells were rinsed with PBS, fixed by paraformaldehyde (Sigma-Aldrich) and permeabilized using 0.1% Triton X-100. Terminal deoxynucleotidyl transferase with fluorescein was added on the slides for 60 min. The slides were immersed three times with PBS. To visualize nuclei, DAPI (Invitrogen) was added before observation. Apoptotic cells were counted with the presence of green fluorescence under fluorescence microscopy. Cell-in-cell apoptotic rate was calculated as followed:


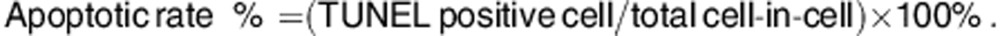


### Lysotracker labeling

Lysotracker red (Invitrogen) was diluted as 1 : 10 000 and added into the cell culture medium for 30 min. Cells were washed three times with PBS and fixed with paraformaldehyde for 20 min. Lysotracker Red-positive cells were counted under fluorescence microscope.

### ROS labeling

Intracellular ROS levels were measured using 2,7-dichlorodihydrofluorescein diacetate (H_2_DCFDA) (Sigma-Aldrich). Briefly, tumor cells were seeded on coverslips in 24-well plates. Twelve hours later (40–70% confluence), immune cells were added to the tumor cells and cultured for certain times. After the coculture, tumor cells on coverslips were washed with PBS, treated with 100 *μ*M H_2_DCFDA in 1% FBS–DMEM at 37 °C, 5% CO_2_ for 1 h. Cells were then washed with PBS, observed and counted under fluorescence microscope (Carl Zeiss).

### Transmission electron microscopy

For transmission electron microscopy (TEM) observation, cells were fixed in 0.1 M PBS (pH7.4) containing 1% glutaraldehyde and 2% paraformaldehyde at 4 °C for 2 h and postfixed in 0.1% PBS with 1% osmiumtetroxide. After dehydration through the gradient ethanol solutions, the specimens were embedded in Araldite-Epon (Embed-812, Electron Microscopy Sciences, Hatfield, PA, USA). Ultrathin sections were prepared with an ultramicrotome (Leica, Bensheim, Germany). Pale-gold sections were collected on 200 mesh copper grids. Ultrathin sections were stained with uranyl acetate and lead citrate (Electron Microscopy Sciences) and examined with an electron microscope (Hitachi, Tokyo, Japan).

### Western blotting

For the detection of GzmB in immune cells, cells were collected and lysed in RIPA buffer (Shanghai Biocolor BioScience Technology Company, Shanghai, China) and centrifuged at 13 000 r.p.m. at 4 °C for 10 min. Protein concentrations were determined by colorimetric assay. Equal amounts of proteins (20 *μ*g) were separated by 10% SDS-polyacrylamide gel electrophoresis and electro-transferred to nitrocellulose membranes. Membranes were blocked with 5% BSA powder in Tris-buffered saline (pH7.4, 0.01 M) containing 0.1% Tween-20 (TBST) for 1 h at room temperature, then incubated with antibodies against tubulin (Invitrogen) and Gzm B (Abcam) overnight at 4 °C. After washing three times with TBST, the membranes were incubated with HRP-conjugated goat anti-rabbit IgG at room temperature for 1 h. Protein bands were visualized using enhanced chemiluminescence (ECL) reagent (Thermo Fisher Scientific, San Jose, CA, USA).

### Cytoxicity assay

Five thousand tumor cells (target cells) per well were seeded in triplicate in 96-well plates and cultured for 24 h. Immune cells (effector cells) were cocultured with target cells at certain effector-target (E:T) ratios as indicated for 4 h. The medium was discarded after incubation and the effector cells were removed by gently shaking. Hundred microlitres per well of CCK-8 reaction reagent (1 : 10 dilution in culture medium) (DojinDo Lab, Tokyo, Japan) was added for another 2 h incubation. Measure the absorbance at 450 nm using a microplate reader (Bio-Tek, Instruments, Inc., Winooski, VT, USA). Cytotoxity of immune cells were calculated as follows:


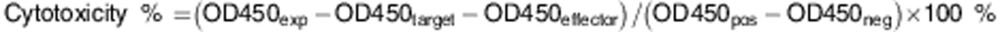


Where OD450_exp_: the mixture of target and effector cells; OD450_target_: target wells only; OD450_effector_: effector wells only; OD450_pos_: target cells treated by 0.2% Triton X-100; OD450_neg_: culture medium only.

### Statistical analysis

The data were presented as means±S.E.M. Statistical analysis was performed using the unpaired two-tailed Student's *t*-test and the Welch's *t*-test by StatView 5.0 software (SAS Institute, Cary, NC, USA). *P*<0.05 was considered statistically significance. All the experiments were repeated at least three times.

## Figures and Tables

**Table 1:** Relationship between cytotoxicity of immune cells and cell-in-cell death manner

**Internalized cell**	Cytotoxic activity*	**Target cell**	**Cell-in-cell death manner**
*Tumor cell lines*			
A431	/	A431	Homotypic entosis
MCF7	/	MCF7	
HCT8	/	HCT8	
HepG2	/	HepG2	
			
*Immune cells*			
Primary immune cells			
CD4^+^T cell	20.8%	MCF7	Heterotypic emperitosis
CD8^+^T cell	48.5%		
CIK	47.5%		
LAK	35.25%		
CD56^+^NKcell	31.6%		
CD19^+^ B cell	7.4%		Heterotypic entosis
CD14^+^ Monocyte	3.2%		
			
*Immune cell-derived cell lines*			
NK92	36.6%		Heterotypic emperitosis
CCRF	2.6%	MCF7	Heterotypic entosis
RAJI	2.8%		

Abbreviations: CIK, cytokine-induced killer cells; LAK, lymphokine-activated killer cells; PBMCs, peripheral blood mononuclear cells

Data were obtained from PBMCs of 10 donors separately

## Abbreviations

ROS: reactive oxygen species
GzmB: granzyme B
LC3: microtubule-associated protein 1A/1B-light chain 3
CIK: cytokine-induced killer
LAK: lymphokine-activated killer
LAMP1: lysosomal-associated membrane protein 1
NAC: N-acetyl-L-cysteine
Cyt c: cytochrome c
